# Developmental Expression of the Nfe2-Related Factor (Nrf) Transcription Factor Family in the Zebrafish, *Danio rerio*

**DOI:** 10.1371/journal.pone.0079574

**Published:** 2013-10-24

**Authors:** Larissa M. Williams, Alicia R. Timme-Laragy, Jared V. Goldstone, Andrew G. McArthur, John J. Stegeman, Roxanna M. Smolowitz, Mark E. Hahn

**Affiliations:** 1 Biology Department, Woods Hole Oceanographic Institution, Woods Hole, Massachusetts, United States of America; 2 Biology Department, Bates College, Lewiston, Maine, United States of America; 3 Andrew McArthur Consulting, Hamilton, Ontario, Canada; 4 Department of Biology and Marine Biology, Roger Williams University, Bristol, Rhode Island, United States of America; Deakin School of Medicine, Australia

## Abstract

Transcription factors in the CNC-bZIP family (NFE2, NRF1, NRF2 and NRF3) regulate genes with a wide range of functions in response to both physiological and exogenous signals, including those indicating changes in cellular redox status. Given their role in helping to maintain cellular homeostasis, it is imperative to understand the expression, regulation, and function of CNC-bZIP genes during embryonic development. We explored the expression and function of six *nrf* genes (*nfe2*, *nrf1a*, *nrf1b*, *nrf2a*, *nrf2b*, and *nrf3*) using zebrafish embryos as a model system. Analysis by microarray and quantitative RT-PCR showed that genes in the *nrf* family were expressed throughout development from oocytes to larvae. The spatial expression of *nrf3* suggested a role in regulating the development of the brain, brachia and pectoral fins. Knock-down by morpholino anti-sense oligonucleotides suggested that none of the genes were necessary for embryonic viability, but *nfe2* was required for proper cellular organization in the pneumatic duct and subsequent swim bladder function, as well as for proper formation of the otic vesicles. *nrf* genes were induced by the oxidant tert-butylhydroperoxide, and some of this response was regulated through family members Nrf2a and Nrf2b. Our results provide a foundation for understanding the role of *nrf* genes in normal development and in regulating the response to oxidative stress in vertebrate embryos.

## Introduction

The nuclear factor-erythroid-2 (NFE2)-related factor (*NRF*) genes encode transcription factors in the Cap’n’Collar basic leucine zipper (CNC-bZIP) gene family. Members of this family include NFE2 [1,2], NRF1 (NFE2L1) [3,4], NRF2 (NFE2L2) [5], and NRF3 (NFE2L3) [6]. (For nomenclature conventions, see footnote 3 of ref [7]..) These transcription factors occur in vertebrates from fish [8] to mammals [9,10], and are known to regulate metabolic [11,12], cell cycle [12-14] and cytoprotective processes [9,13,15-18]. In mice, Nrf proteins have been shown to play important and often essential roles in development. *Nfe2*-null mice lack circulating platelets [19], the *Nrf1* knockout is embryonic lethal [20], *Nrf2*-null mice are highly sensitive to carcinogens and oxidative stress [21], and the *Nrf3* knockout does not show abnormalities under normal conditions but is susceptible to lymphoma induced by exposure to carcinogens [22]. Although studies in mice have provided important information about the function of Nrf genes, viviparous development makes it difficult to directly observe the role and effects of genes throughout the embryonic period. 

Zebrafish are a powerful model for studying molecular mechanisms of vertebrate development. Genes and signaling pathways [23,24] and molecular mechanisms of developmental toxicity [25-29] are highly conserved between fish and mammals. Furthermore, due to the whole genome duplication that occurred during early teleost radiation [30-33], zebrafish have duplicated copies (paralogs) that are co-orthologs of many single copy human genes [30,34]. Paralogs that have undergone subfunctionalization provide an opportunity for new mechanistic insights and thus new understanding about the functions of their single human counterpart [32,35]. In zebrafish, there are several paralogs within the *nrf* gene family, including *nrf1a* and *nrf1b* as well as *nrf2a* and *nrf2b* [7]. In total, zebrafish have six *nrf* family genes. 

In mammals, NFE2 forms a heterodimeric transcription factor with small MAF proteins, regulates expression of globin genes [2,36], and may regulate the oxidative stress response in mature erythrocytes [17]. In zebrafish, *nfe2* is a single copy gene [8]; its duplicate appears to have been lost [7]. In embryos *nfe2* is highly expressed in the intermediate cell mass during erythroid differentiation [8]. Apart from *in situ* hybridization during development and protein characterization, the expression and role of *nfe2* in development are largely unexplored. 

NRF1 in mammals is expressed in many tissues [4]; it plays a role in regulating redox balance in the liver [37] and in regulating genes involved in development [20], oxidative stress [38,39], cytoskeletal organization [40], and the proteasome [41]. In response to UVB damage, mammalian NRF1 regulates nucleotide excision repair and glutathione homeostasis, and may act as a tumor suppressor [13]. The two zebrafish *nrf1* paralogs, *nrf1a* and *nrf1b*, are syntenic with the duplicated *hoxb* clusters, similar to the human NRF1 that is near the human HOXB cluster [7]. Prior to the current study, the only information about expression of zebrafish *nrf1* had been from EST data [38] and a limited assessment by qualitative RT-PCR [7].

NRF2 has a broad tissue distribution in mammals and acts as a pleiotropic transcription factor involved in regulating the susceptibility to disease, in mediating the response to xenobiotic exposure and oxidant stress, and in proteome maintenance [42]. In zebrafish, two paralogs (*nrf2a* and *nrf2b*) are broadly expressed both in the embryo and adult and apparently have undergone subfunctionalization [7]. The primary role of *nrf2a* seems to be in the regulation of cytoprotective genes upon oxidative stress [7,9]. Conversely, *nrf2b* is a negative regulator of embryonic gene expression both basally and under oxidative stress conditions [7]. 

NRF3 has been found to be expressed at high levels in mammalian placenta and the B cell lineage and at lower levels in the heart, lung, kidney, and pancreas [6]. NRF3 may play a protective role in regulating genes responding to oxidative stress, as Nrf3-deficient mice treated with an oxidant suffer acute lung damage [43]. Zebrafish have one *nrf3* ortholog, and like *nfe2*, the duplicated gene appears to have been lost [7]. The expression and function of *nrf3* in zebrafish was completely unexplored prior to this study. 

In this paper, we address fundamental aspects of the expression and role of six *nrf* genes during vertebrate development using the zebrafish as a model system. We show that the expression of *nrf* genes varies temporally throughout development and that *nrf* genes are induced following a pro-oxidant exposure and are potentially cross-regulated by gene family members Nrf2a and Nrf2b. We also identify a novel role for *nfe2* in the cellular organization of epithelia in the pneumatic duct. 

## Experimental Procedures

### Fish husbandry

Zebrafish of the Tupfel/Longfin mutation (TL) wild-type strain were used in all experiments. Adults were maintained and embryos were collected as previously described [44]. This study was carried out in strict accordance with the recommendations in the Guide for the Care and Use of Laboratory Animals of the National Institutes of Health. The protocol was approved by the Woods Hole Oceanographic Institution Animal Care and Use Committee (Animal Welfare Assurance Number A3630-01). 

### Cloning and confirmation sequencing of *nrf* genes

In order to determine *nrf* cDNA sequences in the TL wild-type strain, and identify strain-specific polymorphisms, *nrf* cDNAs were amplified with primer pairs (Table 1), using Advantage cDNA Polymerase. The cycling conditions were [94°C, 1 min], [94°C, 30 sec; 68°C, 3 min] for 35 cycles, followed by 68°C for 3 minutes and 10 minutes at 15°C. The PCR products were purified with the GENECLEAN kit (Qbiogene, Quebec, Canada), ligated into pGemT Easy Vector System (Promega, Madison, WI) and constructs were sequenced. Prior to confirmation of *in vitro* expression, gene products from pGemT were subcloned into pcDNA3.1/Zeo (+) (Invitrogen, Carlsbad, CA). Sequences of clones were deposited into Genbank and have accession numbers JX867113-JX867116.

**Table 1:** Primers used for gene amplification, cloning, and qPCR of *nrf* genes.

**Gene**	**Primer sequences (5’-3’)**	**Amplicon size**	**T (°C)**	**Ref**
*nfe2*	F-TTCTGGTCTGAACTCCTTGCTGTGG	1557	68	-
	R-GGTGAACTATCACTTTAATCAAACAT			
*nrf1a*	F-CAGTTCGCACGCCCTTATTTACTGAC	2376	68	-
	R-GCTGATGGACTTAACAGCAGACAG			
*nrf1b*	F-CGTAACCTAATTTGGTTTGACG	2885	68	-
	R-GTCCTCCTTGACTTCCCATATC			
*nrf3*	F-AAATTGAGCAGTTGCTCCCCTCC	2346	68	-
	R-CTCACCTCAAAGATAAAACTCACC			
*nfe2*-qPCR	F-CAGAGTTTGAGGAACCCAATGAG	127	57	-
	R-CACAAGTGGCTGGAATGGATTC			
*nrf1a*-qPCR	F-CCAGAGTTGACAGGTCCTGG	158	64	-
	R-CATAACCTGTGATTCCATGATAGAC			
*nrf1b*-qPCR	F-GCAGGACATGGAGGTGAACAATACG	120	66	-
	R-GGATCGTGGGAGCCCAAAATTTCC			
*nrf2a*-qPCR	F-GAGCGGGAGAAATCACACAGAATG	82	65	[7]
	R-CAGGAGCTGCATGCACTCATCG			
*nrf2b*-qPCR	F-GGCAGAGGGAGGAGGAGACCAT	269	68	[7]
	R-AAACAGCAGGGCAGACAACAAGG			
*nrf3*-qPCR	F-GCATGAGGATTTAGTGGTTAGTGG	108	64	-
	R-GGAGTCAAAATCATCAAAGTCAG			
*actb1*-qPCR	F-CAACAGAGAGAAGATGACACAGATCA	140	65	[87]
	R-GTCACACCATCACCAGAGTCCATCAC			

Primer sequences are written 5’-3’ and amplicon size and melting temperature (T) are shown. For primers used previously, a reference is cited. Accession numbers for sequences newly reported here are: *nrf1a* (JX867114), *nrf1b* (JX867116), *nrf3* (JX867113), *nfe2* (JX867115).

### Sampling, Chemical exposure, RNA extraction, and cDNA synthesis

#### Embryos for Developmental Series

As described in Timme-Laragy et al. [7], 4 pools of 30 staged embryos from a single clutch were reared at low density at 28.5°C in 0.3x Danieau’s and flash frozen in liquid nitrogen at 6, 12, 24, 48, 60, 72, 96, and 120 hpf and stored at -80°C. Hatched and unhatched embryos were collected separately at the 48 and 60 hpf time points. Unfertilized eggs, representing the 0 hpf time point, were manually stripped from 3 females, combined, and flash frozen as 3 separate biological replicates and stored at -80°C. Although it limited our ability to determine individual variation amongst embryos, pooling of embryos was needed to provide sufficient amounts of RNA for measuring expression of multiple genes. 

#### Chemical exposure of embryos to tBOOH

Embryos (3 pools of 30 staged embryos) were exposed to tert-butylhydroperoxide (tBOOH). Embryos (96 hpf) were exposed to 0, 0.5 or 0.75 mM tBOOH for 6 hours in 150x20mM glass petri dishes. Following tBOOH exposure, embryos were immediately placed in RNAlater and stored at -80°C.

#### RNA extraction and cDNA synthesis

Total RNA was isolated from pooled embryo samples using RNA STAT-60 (AMS Biotechnology, Abingdon, UK) following the manufacturer’s protocol. cDNA was synthesized from 1 µg of total RNA using random hexamers and the Omniscript cDNA Synthesis Kit (Qiagen, Valencia, CA). 

### Quantitative real-time RT-PCR gene expression profiling

Using the MyiQ Single-Color Real-Time PCR Detection System (Bio-Rad, Hercules, CA), QPCR was conducted with the iQ SYBR Green Supermix (Bio-Rad) on *nrf* genes. For each sample, duplicate reactions in separate wells were run containing 5 ng of cDNA. The PCR conditions were 95°C for 3.5 minutes followed by 35 cycles of 95°C for 15 seconds and 25 seconds at a gene specific temperature. Gene-specific primers and temperatures are listed in Table 1. Following each run, a melt curve was generated to ensure the amplification of single product. All primers were tested for amplification efficiency using a calibration dilution curve and slope calculation approach [45]. β-actin 1 (*actb1*) was chosen as a housekeeping gene due to its limited variation in expression with embryonic development and chemical exposure [46]. We confirmed the suitability of this housekeeping gene with the set of samples used in this study. Expression of genes was analyzed using the comparative ∆∆C_T_ method [47].

### Statistical analysis of QPCR data

#### Developmental Series

Data were analyzed with Microsoft Excel using the ∆∆C_T_ method to calculate fold change. Values are presented as mean ± SEM, and *N* is defined as the number of pools of embryos. Relative expression was normalized to the 120 hpf value within a gene. As described in Timme-Laragy et al. [7], Statview for Windows (version 5.0.1; SAS Institute, Cary, NC) was used to determine differences in expression due to hatching state. Data were log-normalized and six statistical outliers were removed from the *nrf2b* development series (one data point from each of six time points: 0, 6, 24, 48 (unhatched), 48 (hatched), and 96 hpf). If an ANOVA yielded significance (*p* < 0.05), Fisher's protected least significant differences test was used as a *post hoc* test with Bonferroni correction.

#### Chemical Exposure and Nrf2a and Nrf2b crosstalk

Data were analyzed with Statview for Windows (version 5.0.1; SAS Institute, Cary, NC) and presented as mean ± SEM where *N* is defined as the number of pools of embryos. Significance in ANOVA was determined with a *p* ≤ 0.05. When significance was yielded, Fisher’s protected least-significant differences test was used as a *post hoc* test with Bonferroni correction. Relative expression was normalized to the water control for tBOOH. For Nrf2a and Nrf2b morpholino data, relative expression of each gene was normalized to the water control with control morpholino.

### Microarray gene expression profiling

Zebrafish embryo rearing, sampling, and microarray analysis were performed as previously reported [29]. Briefly, four replicate pools of 100 embryos were examined at 3, 6, 12, 24, 36, and 48 hpf. Agilent 4 × 44k DNA gene expression microarrays (part #GPL11077, WHOI Zebrafish 44k v1.0 custom-commercial Chemical Defensome array, Agilent Technologies, Santa Clara, CA), which included custom probes to ensure adequate coverage of the chemical defensome [29], were used to examine gene expression levels. Single color microarray data (Cy3) was normalized using the non-linear scaling method of Schadt et al. [48], where saturated probes and probes not above background in all replicated were removed. Because standard ANOVA is not appropriate for these data due to auto-correlation between time points, we used Bayesian Estimation of Temporal Regulation (BETR; [49]) to analyze the developmental time series. Normalized Cy3 values for each probe were log transformed, median-centered, and analyzed using BETR relative to 3 hpf. Normalized average Cy3 signals were compared for genes of interest among time points. All probes reported here had significant differential expression among sampling times. The microarray data have been deposited into the Gene Expression Omnibus (GEO) database with NCBI GEO accession number GSE24840.

### In situ hybridization

*In situ* hybridization of *nrf3* on whole embryos was performed using established procedures [50,51]. Sense and anti-sense *nrf3* RNA probes were created by restriction digest of the *nrf3* construct (in pGemT) with XhoI, yielding a 1080 bp sense probe and 1328 bp anti-sense probe. The *nrf3* probe covers a 1240-bp region of the 3’ end of the cDNA and includes 87 base pairs of the 3’ UTR. In designing the probe, we carefully considered possible sequence identity with other *nrf* transcripts. Nucleotide sequence identities between *nrf3* and other *nrf* transcripts in the region covered by the probe were as follows: 45% with *nfe2*, 47% with *nrf1a*, 51% with *nrf1b*, 49% with *nrf2a*, and 45% with *nrf2b*. The overlap between the *nrf3* probe and other *nrf* transcripts was typically interspersed throughout the sequence with no long runs of overlap (mainly, 4-5 nt at a time). We also conducted a Blast search using the *nrf3* probe against the zebrafish genome; no significant overlap was found other than the *nrf3* gene itself. Probes were labeled with digoxigenin-UTP by *in vitro* transcription (Roche San Francisco, CA) with either T7 (sense) or SP6 (anti-sense) polymerase. Pre-hybridization was carried out for 2 hours at 65°C. Hybridization was carried out overnight with 300 ng of probe per pool of seven embryos at a concentration of approximately 1.5 µg/mL. Embryos were blocked for 4 hours at room temperature and incubated with anti-digoxigenin alkaline phosphatase conjugated antibody (Roche, San Francisco, CA) at 4°C overnight. Embryos were imaged by light microscopy (Axio Observer.A1, Carl Zeiss, Oberkochen, Germany).

### Morpholino oligonucleotides

Morpholino antisense oligonucleotides (MO) were designed to block initiation of translation of zebrafish *nfe2*, *nrf1a*, *nrf1b* and *nrf3* and obtained from Gene Tools, LLC (Philomath, OR). The first *nfe2* MO (AGTTCCTGCCAGGCCAAGTCCATCT) complements two residues of the 5’UTR, the start codon (underlined), and 20 residues of the coding region. A second, non-overlapping *nfe2* MO (AACGATGTGTCCGTAATCCAGTGAC) complements 25 residues of the 5’UTR and targets a region whose 3’ end is 15 residues upstream of the sequence targeted by the first *nfe2* morpholino. The *nrf1a* MO (ATGGCCCAAACCATCACCGGCAGCA) complements 11 residues of the 5’UTR, the start codon (underlined), and 11 residues of the coding region. The *nrf1b* MO (AATCACGCAAACAAACGTCAAACCA) complements 25 residues of the 5’UTR and its 3’ end is located 34 residues upstream of the start codon. The *nrf3* MO (TTTTAACCTCAGGAGGCTTAAACGA) complements 25 of the 5’UTR and its 3’ end is located 14 residues upstream of the start codon. The standard control-MO from Gene Tools was also used (CCTCTTACCTCAGTTACAATTTATA). All MO were tagged at the 3’ end with fluorescein in order to detect successful injection and incorporation of MO into embryos. MOs targeting *nrf2a* and *nrf2b* are previously described [7].

### Confirmation of MO translation inhibition by *in vitro* protein synthesis

To confirm the efficacy of morpholinos to inhibit translation of Nrf proteins, the TnT T7 Quick Coupled Reticulocyte Lysate System (Promega, Madison, WI) was used to synthesize [^35^S]methionine-labeled zebrafish NF-E2, Nrf1a, Nrf1b and Nrf3 protein as per manufacturer’s protocols and as described previously [52]. Briefly, the TnT reaction included 1 μl of [^35^S]methionine (> 1000 Ci/mmol at 10 mCi/ml) and 2 μl of the respective *nrf* cDNA in pcDNA3.1/Zeo (+) (0.5 μg/μl) in a final volume of 25 μl with H_2_O. For reactions containing MOs, 0.5 μl of a 25 μM stock of the appropriate MO was added, resulting in a final concentration of 500 nM. Labeled protein products were resolved by SDS-PAGE, dried on Whatman filter paper, and visualized on film. To quantify the reduction in protein products, gel fragments containing a single protein band were excised and placed into 7 mL glass scintillation vials containing 5mL ScintiVerse II cocktail (Fisher Scientific, Pittsburg, PA). Counts were carried out for 5 minutes on a Beckman Coulter LS6500 Multi-purpose Scintillation counter (Brea, CA) and background was subtracted. 

### Microinjection of zebrafish embryos with morpholinos

Zebrafish embryos at the two- to four-cell stage were injected with 2.5-5 nL of a 0.1 mM solution of MO targeting *nfe2*, *nrf1a*, *nrf1b*, *nrf2a*, *nrf2b*, *nrf3*, or a control-MO, using a Narishige IM-200 microinjector (Tokyo, Japan). Injection volumes were calibrated as described previously [52]. The injections resulted in delivery of MO amounts ranging from 2-4 ng. At six to nine hpf, embryos were sorted and screened for successful fertilization and fluorescence. When no developmental phenotypes were observed, the concentration of the morpholino stock was increased to 0.15 mM, keeping the injection volume constant. MO-injected embryos were held in 0.3x Danieu’s solution. At 4 dpf, swim bladder inflation was scored as described earlier [53].

### Histological analysis of the swim bladder

Four-day-old larvae from embryos injected with control morpholino (swim bladder inflated) or *nfe2* morpholino (swim bladder not inflated) were fixed in 4% formaldehyde in 1x phosphate buffer, dehydrated in ethanol and stored in 75% ethanol until embedding. Larvae were sent to Environmental Pathology Laboratories (Sterling, VA) where they were embedded laterally into either Technovit 7100 (Heraeus Kulzer, Hanau, Germany) or paraffin. Sagittal sections were made serially every 2 µm. Sections were mounted on superfrost glass slides and stained with hematoxylin and eosin. Histopathology was conducted by an Olympus BX 40 with an Olympus DP25 camera system. 

### Analysis of blood flow

Transgenic *gata1* ^dsRED^ embryos [54] (15 individuals per treatment) were injected with control or *nfe2-*MOs at one- to four-cell stages. As an index of blood flow, the number of red blood cells passing through the mesencephalic vein (MsV) per 15 s was determined by orienting 48 hpf zebrafish embryos laterally as described by Kubota et al. [55]. Blood flow was recorded with an AxioCam MRc5 camera on a Zeiss Axiovert 200 inverted microscope and analysis (counting of red blood cells) was performed using ImageJ (http://rsbweb.nih.gov/ij/). 

### Analysis of otic vesicles

AB embryos were injected at one- to four-cell stages with control-MO or *nfe2-MO*-1. At 30, 48 and 72 hpf, otic vesicles were imaged with an AxioCam MRc5 camera using a Zeiss Axiovert 200 inverted microscope. 

### *In silico* Promoter Analysis and motif searches

To determine whether Nrf2a or Nrf2b are directly involved in the transcriptional regulation of other *nrf* family members, *in silico* promoter analysis was carried out for AREs and XREs using a fuzzy search algorithm, fuzznuc [56]. 10,000 base pairs upstream of the start site and the entire length of the gene were examined for putative zebrafish AREs (TGA(G/C)nnnTC [57]) and XREs (KNGCGTG [58]). Additional searches were carried out for Nfe2 binding sites [59] determined from ChIP-Seq studies. The zebrafish genome was searched for Nfe2 binding sites in the 10 kb upstream of the start codon of all known genes using a position-specific scoring matrix (PSSM) based on the empirical ChIP-Seq results of Wang et al [59] and the FIMO algorithm of the MEME/MAST package [60](p < 0.0001), with correction for background nucleotide frequencies of complete nuclear genome sequences.

## Results

### Developmental expression profiling

**Figure 1:**
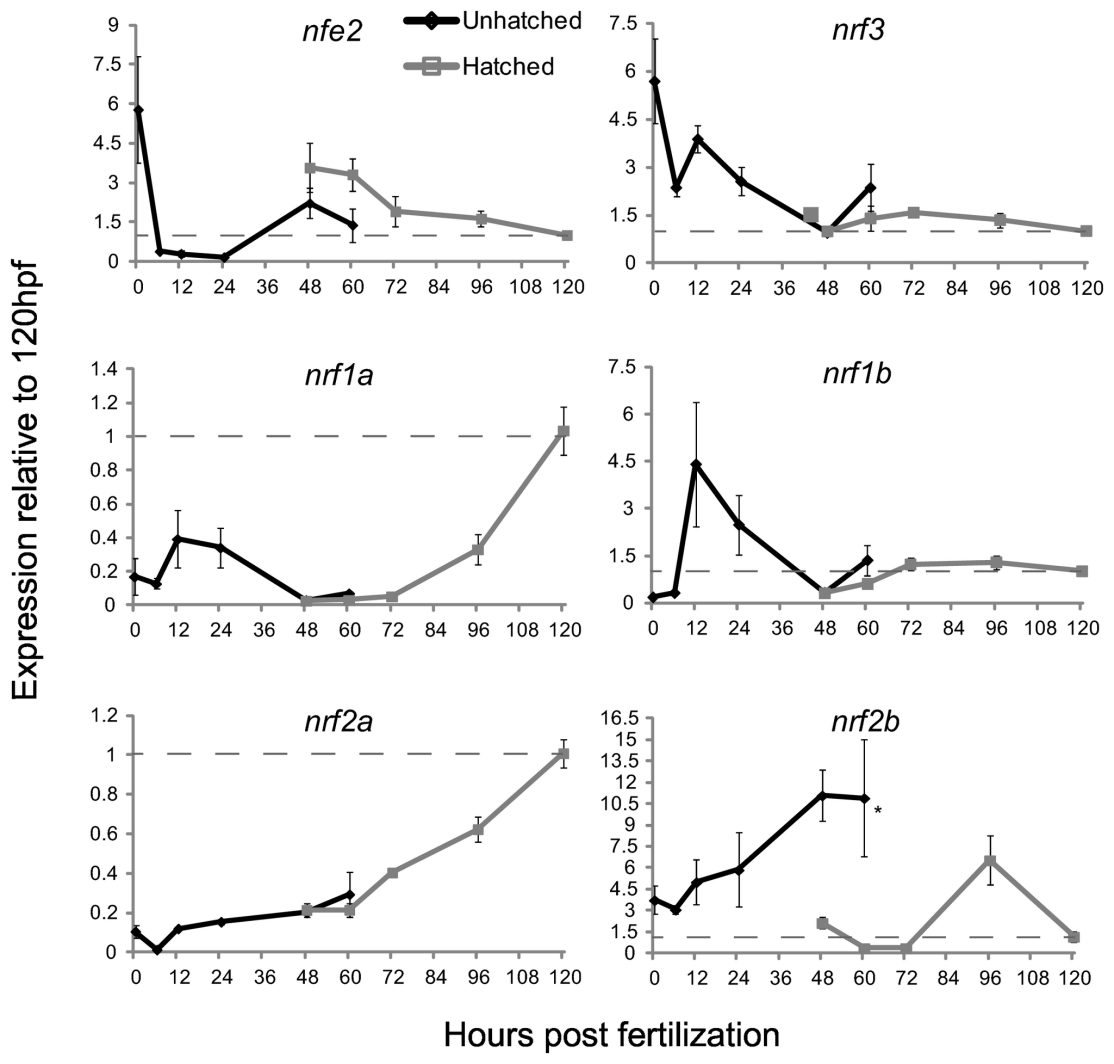
The expression of the *nrf* gene family during development as measured by quantitative real-time PCR. Black lines indicate expression pre-hatch and grey lines indicate expression post-hatch. Values were normalized to the 120 hpf time-point and *β-actin 1* (actb1) was used as the housekeeping gene. Data are presented as the mean ± S.E.M. (error bars), and *N* = 4 pools of 30 embryos. Differences in expression between hatching state were assessed using ANOVA followed by Fisher's PLSD (*, *p* ≤ 0.05). Data for expression of *nrf2a* and *nrf2b* are from Timme-Laragy et al. [7] and are used with permission of the American Society for Biochemistry and Molecular Biology. Previously these data were presented as molecule number. Here we have normalized the data to β-actin 1 expression and to the 120 hpf time point and re-plotted for comparison to the new data on expression of other *nrf* family genes. Dashed lines indicate the value of 1.0 from the 120 hpf time point.

To begin to understand the role of the *nrf* genes during development, their expression was profiled from 0 to 120 hpf using qRT-PCR (Figure 1). Because the data were normalized within each gene, expression values can be compared at different times for each gene but not across genes. *nfe2* transcripts were maternally deposited, but levels fell substantially at 6 hpf and did not increase again until 48 hpf. Expression remained relatively constant from 48 to 60 hpf and then decreased steadily until 120 hpf. Like *nfe2*, *nrf3* was maternally deposited and decreased by 6 hpf, but transcript levels increased again at 12 hpf. Expression decreased between 12 and 48 hpf, but then remained steady. *nrf1a* was expressed from 0-24 hpf but levels were reduced from 48-72 hpf, after which expression increased at 96 and 120 hpf. Conversely, *nrf1b* expression peaked between 12 and 24 hpf and then declined and remained relatively low from 48 to 120 hpf. Hatching did not have a significant effect on expression of *nfe2*, *nrf3*, *nrf1a* or *nrf1b*. For comparison, Figure 1 also includes data for expression of *nfr2a* and *nrf2b*, originally presented (in a slightly different format) by Timme-Laragy et al. [7]. These results showed that *nrf2a* expression increased steadily during development. The expression of *nrf2b* also increased through 48 hpf, but there was a significant effect of hatching on *nrf2b* expression, when expression decreased in hatched embryos compared to unhatched embryos of the same developmental age. 

**Figure 2:**
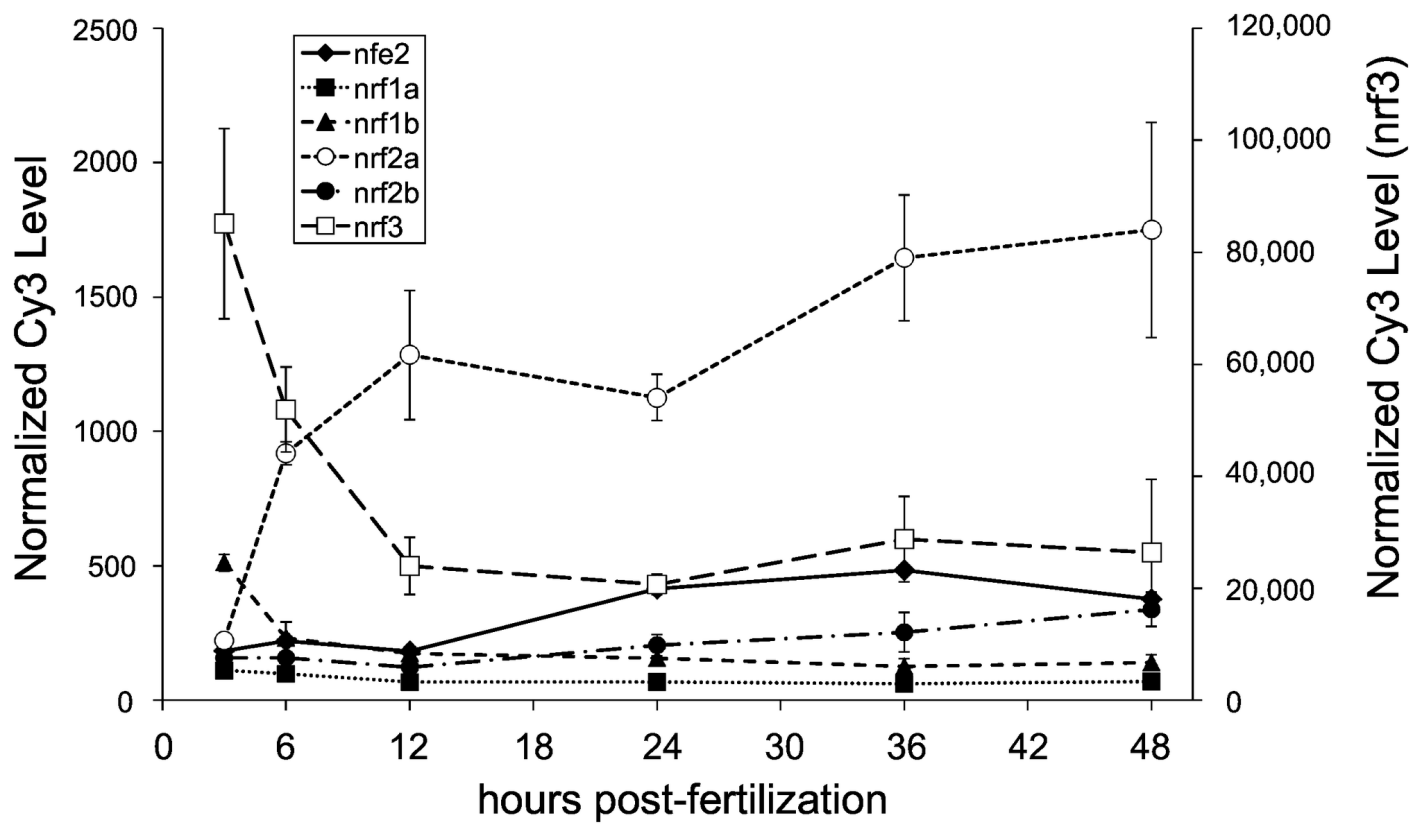
The expression of the *nrf* gene family during development as measured by microarray analysis. Data are presented as normalized Cy3 levels, with average values for *nfe2*, *nrf1a*, *nrf1b*, *nrf2a*, *and* *nrf2b* displayed on the primary (left) y-axis. Values for *nrf3* are displayed on the secondary (right) y-axis. Each time point represents the average normalized value ±SD of 4 replicates, each from 100 embryos. The Agilent probes from which these data were obtained are: *nfe2* (A_15_P109440), *nrf1a* (A_15_P110831), *nrf1b* (A_15_P116909), *nrf2a* (CUST_121_PI358351581), *nrf2b* (CUST_122_PI358351581)*, nrf3* (A_15_P116878). An additional probe for *nrf2a* (A_15_P109504) was present on the array and produced results very similar to those shown here for CUST_121_PI358351581. The microarray data have been deposited with NCBI GEO accession number GSE24840.

Since molecule numbers were not available from the qRT-PCR data and data were normalized within each gene, a microarray was used to determine the expression of *nrf* genes relative to each other during the first 48 hours of development (Figure 2). Microarray and QPCR analyses were performed on samples obtained from independent experiments. The microarray data did not include a 0 hpf time point, so maternal transcript levels were not determined with this method. Gene expression profiles determined by QPCR and microarray for *nfe2*, *nrf1a*, *nrf2a*, and *nrf2b* displayed similar developmental patterns. The developmental patterns of *nrf3* expression measured by qPCR and microarray were similar, except that the pattern at 6 and 12 hpf was reversed. For *nrf1b*, the peak at 12 hpf measured by QPCR was not evident in the microarray data. 

Normalized Cy3 levels for *nfe2*, *nrf1a*, *nrf1b*, *nrf2a*, and *nrf2b* ranged from 100 to 1,800, whereas values for *nrf3* ranged from 80,000 at 3 hpf to approximately 20,000 at 48 hpf (Figure 2). The *nrf* gene with lowest expression was *nrf1a* followed by *nrf1b*. Their average expression values were about half that of *nfe2*. The expression of *nrf2a* was up to10-fold greater than that of all other *nrf* genes except *nrf3*. 

### Localization of nrf3 expression with ISH

**Figure 3:**
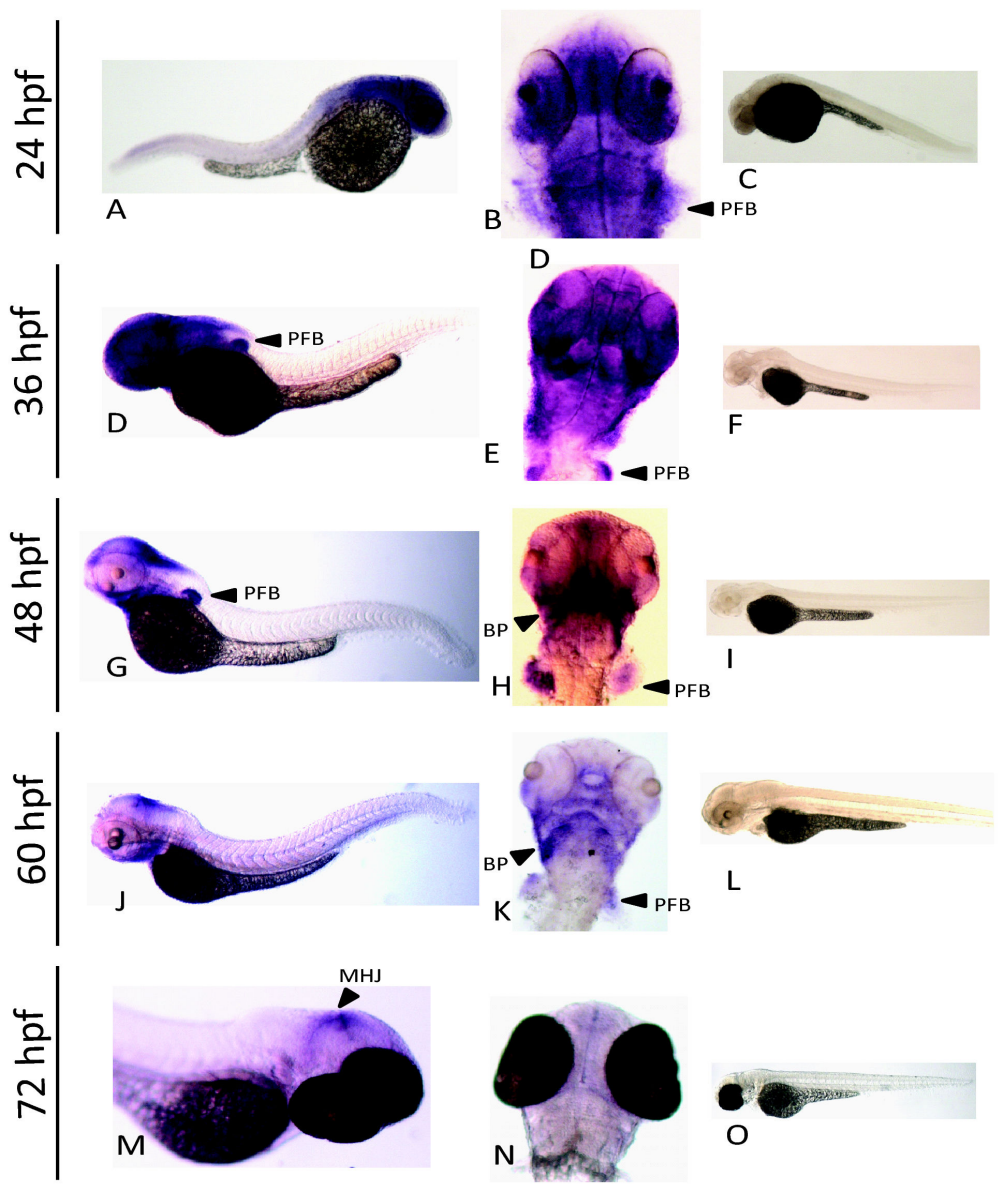
Expression of *nrf3* during development as assessed by whole mount *in* *situ* hybridization. An anti-sense probe was used to determine the spatial expression of *nrf3* at 24 hpf (A,B), 36 hpf (D,E), 48 hpf (G,H), 60 hpf (J,K), and 72 hpf (M,N). Paired sense controls are shown for each time point as well (C,F,I,L,O). Arrows indicate specific features identified by *in* *situ* hybridization: pectoral fin bud (pfb), branchiogenic primordia (bp), and mid hindbrain junction (mhj).

We used *in situ* hybridization to examine the spatial expression of *nrf3*, the *nrf* gene most highly expressed during development; analysis was carried out in 12-hour intervals from 24 to 72 hpf. *In situ* gene hybridization results for *nfe2*, *nrf1a, and nrf2a* have been previously reported [8,61,62]. The most prominent expression of *nrf3* occurred at 24 and 36 hpf; expression was detected in the fore-, mid- and hindbrain as well as in the pectoral fin buds (Figures 3A, 3B, 3D, 3E). The high degree of staining seen is consistent with the relatively high level of *nrf3* expression seen in the array and qRT-PCR data. At 48 hpf and 60 hpf, expression still was seen in the pectoral fin buds, and was less prominent in the forebrain and more concentrated in both the mid- and hindbrain (Figures 3G, 3H, 3J, 3K). Expression was also seen in the branchiogenic primordia. Expression at 72 hpf was almost exclusively in the mid/hindbrain junction (Figure 3M, 3N). There was no signal in sense controls at any time point assayed (Figures 3C, 3F, 3I, 3L, 3O). 

### Effect of *nrf* gene knockdown on development

**Figure 4:**
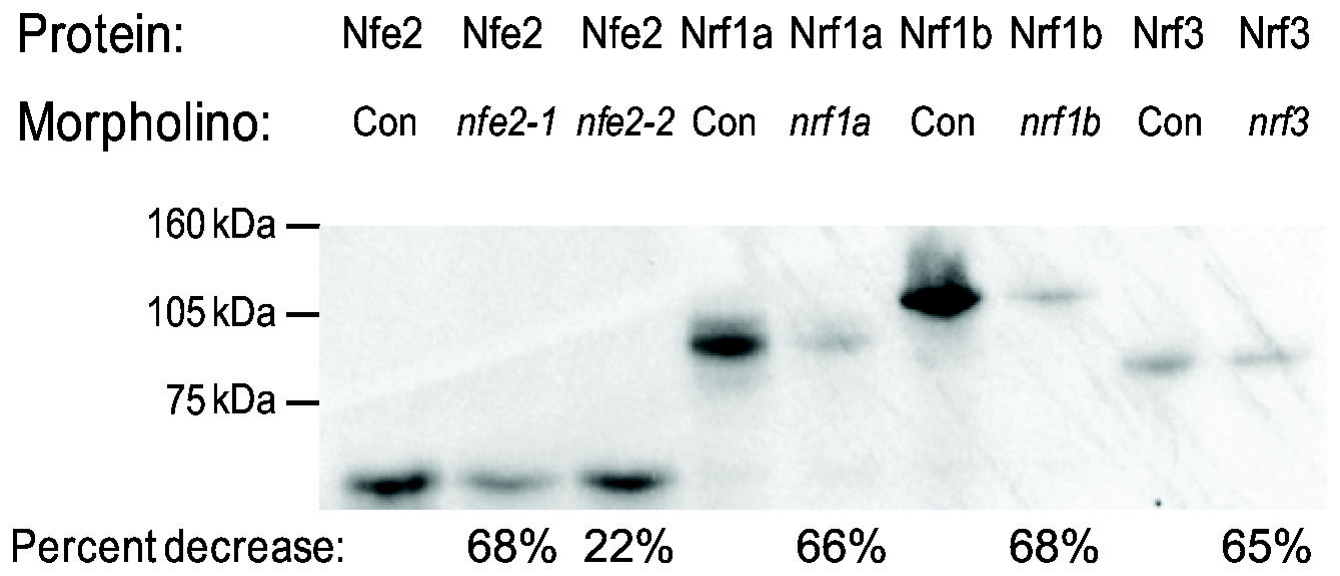
Assessment of morpholino efficacy by *in* *vitro* translation in the presence of [^35^S]methionine. All morpholinos knock down expression (Nfe2 lanes 2,3; Nrf1a lane 5; Nrf1b lane 7; Nrf3 lane 9) and for *nrf1* morpholinos there is specificity for each paralog (data not shown). Scintillation counts, presented as percent decrease over control, give a quantitative measurement of knockdown success.

One approach to identify the importance of *nrf* genes in developmental processes is to knock down their expression in the embryo. The *in vitro* translation-blocking efficiency of *nrf* morpholinos ranged from 65 to 68%, with the exception of morpholino 2 for *nfe2* (22%; Figure 4). To determine whether any morphological abnormalities were associated with the reduction of Nrf proteins, embryos were injected with morpholinos and observed every 12 hours until 120 hpf. No gross morphological abnormalities were observed for control MO, *nrf1a*-MO, *nrf1b*-MO, a combination of *nrf1a*-MO and *nrf1b*-MO, or *nrf3*-MO. Additional experiments were conducted using higher concentration of morpholino (0.15 mM). However, at this concentration off-target effects were observed. Off-target effects observed in more than 50% of the MO-injected embryos included hatching gland deformities, pericardial edema, and bent tail post-hatching.

**Figure 5:**
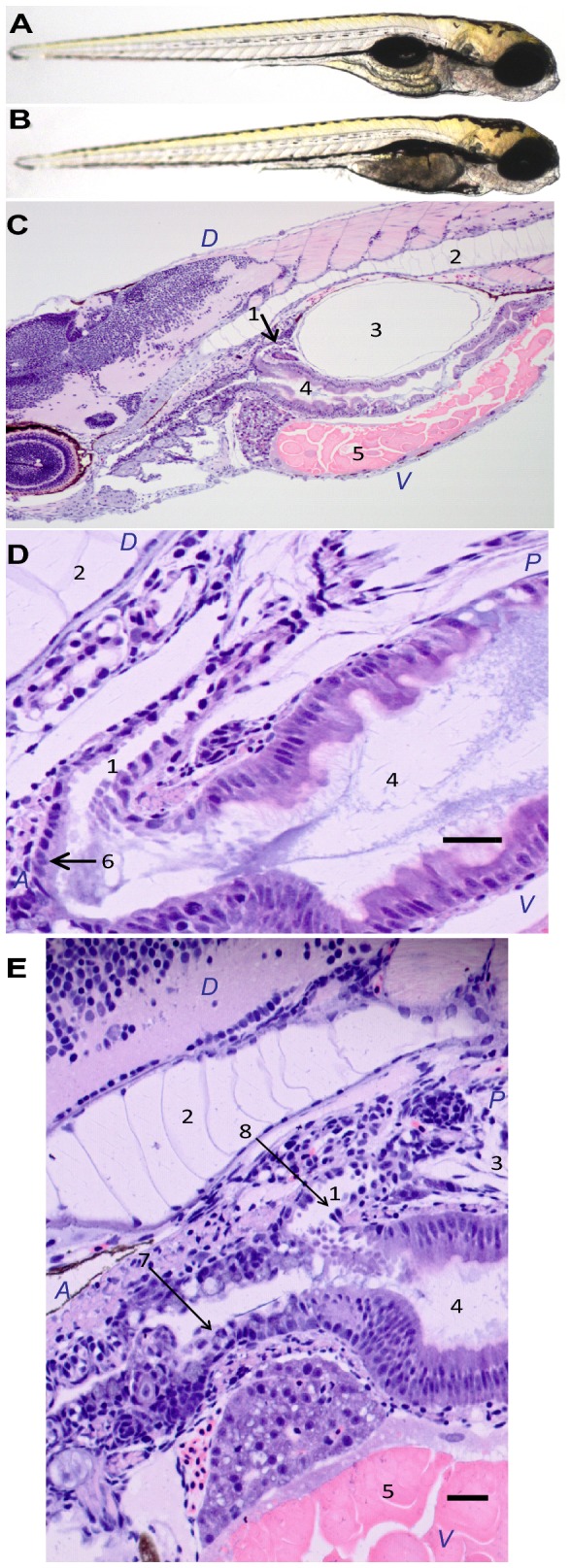
Effects of Nfe2 knockdown on swim bladder inflation and histology. Thirty larvae were sampled at 96 hpf after being injected as embryos with either control morpholino (A) or *nfe2* morpholino (B) and histological analysis was conducted on eight larvae from each treatment.  Normal histology is shown for larvae injected with control MO (C,D).  In larvae with Nfe2 knocked down, cellular disorganization is shown in the mucus cells lining the esophagus (E).  Scale bars represent 25 µm (D) and 30 µm (E). In (C), (D), and (E), cellular structures are numbered as follows: 1: pneumatic duct; 2: notochord; 3: swim bladder; 4: gastrointestinal lumen; 5: yolk; 6: normal columnar epithelium; 7: irregular, disordered epithelium lacking well developed mucus cells; 8: irregular epithelium. Anterior (A), posterior (P), dorsal (D), and ventral (V) are also labeled.

In contrast to the lack of specific phenotypes in embryos injected with MOs targeting *nrf1a, nrf1b*, or *nrf3*, injection of an *nfe2*-MO (0.1 mM) resulted in the failure of the swim bladder to inflate at 96 hpf in 95% of the injected embryos (Figure 5B versus 5A) (Table 2). The specificity of this effect was confirmed using a second, non-overlapping *nfe2* MO that also targeted the region surrounding the translation start site (Table 2).

**Table 2:** Phenotype of *nfe2* morphants.

	**MsV blood flow (cells/15-sec)**	**Swim bladder inflation**	**Otic vesicle abnormalities**
Control-MO	98±2.5 (15)	20/20	0/20
*nfe2-MO*-1	96±2.9 (15)	1/20	20/20
*nfe2-MO*-2	96±2.5 (15)	1/20	Not done

Embryos were injected with morpholino antisense oligos (MOs) at one to four cell stages, with either control MO, *nfe2-MO*-1, or *nfe2-MO*-2, and blood flow through the mesencephalic vein (MsV; 48 hpf), swim bladder inflation (96 hpf), and otic vesicle abnormalities (30, 48, and 72 hpf) were recorded. Each endpoint was assessed in a separate experiment; for details, see Methods. For blood flow measurements, the number of red blood cells passing through the mesencephalic vein (MsV) per 15 s was determined by orienting 48 hpf zebrafish embryos laterally as described in Kubota et al. [55] and recording the blood flow with an AxioCam MRc5 camera. Cell counts were determined using ImageJ. The number of larvae with normal swim bladder inflation and otic vesicle abnormalities are noted for each treatment. Results are expressed as mean ± standard deviation and sample size by numerical values in parentheses, or by a ratio. No significance was found between MsV values for *control-MO*- and *nfe2-MO*-injected embryos as determined by a one-way ANOVA (*p* < 0.05).

Histological analysis of larvae treated with control MO showed the normal pneumatic duct connection between the esophagus and the swim bladder (Figure 5C,D). Epithelial cells along the duct were orderly and cell morphology progressed from columnar at the entrance to the duct to cuboidal to squamous at the entrance to the swim bladder. In larvae in which Nfe2 had been knocked down, the pneumatic duct connection was intact but the number of epithelial cells and the regular progression from columnar to squamous was disrupted (Figure 5E). In the most severe example, the epithelial cells lining the duct were irregular in shape and size, and varied from squamous to mound-like shapes. Cellular disorganization also extended beyond the pneumatic duct. In Nfe2 morphants, there was a loss of well-developed columnar, mucus cells lining the esophagus at the junction of the swim bladder duct. Muscle cells around the junction of the duct with the esophagus appeared normal by histological examination, but were markedly less developed in Nfe2 animals. Swim bladder lumens were present in both Nfe2 morphants and control animals. 

The phenotypic differences noted indicate that the disorganization of the epithelium in the duct, the lack of mucus cells at the ductal entrances and potentially the lack of peri-ductal muscle function most likely disrupted the normal function of swim bladder inflation and air retention. The gill epithelial and cartilage cells, and cartilage in pharyngeal support areas, appeared to be abnormal in development, but were not evaluated in detail for this study. 

**Figure 6:**
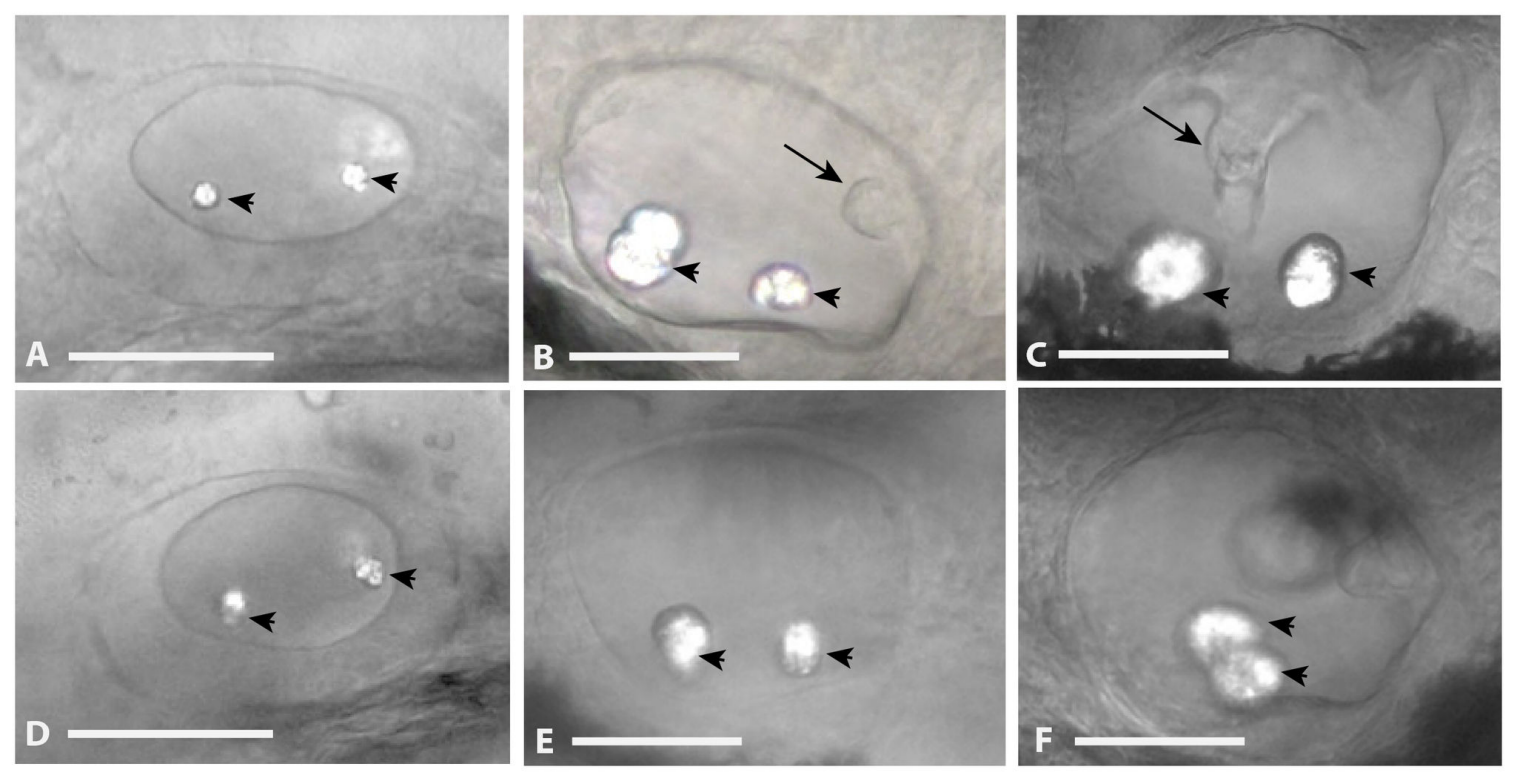
Otic Vesicle Defects in *nfe2* morphants. Otic vesicles were laterally imaged at 30 hpf (panel A, D), 48 hpf (panel B, E), and 72 hpf (panel C, F) following injection of control MO (panels A, B, C) or *nfe2-MO*-1 (panels D, E, F) at the one to four-cell stage. Otoliths are marked with an arrowhead and projections of epithelium forming the semicircular canal system are noted with an arrow in panel B. The fusion of the canal system is noted by an arrow in panel C. Scale bars equal 50 μm.

Because *nfe2* expression has been observed in the otic vesicles at 36 and 48 hpf [8], we looked for effects of *nfe2* knock-down in these structures. Otic vesicles from twenty AB embryos per treatment were imaged following treatment with control-MO or *nfe2-MO*-1 at 30, 48 and 72 hpf. At 30 hpf, there were no observable differences between the otic vesicles or otic vesicle morphology between treatments (Figure 6, panels A and D). The number, size and space between otoliths was similar across all embryos (otoliths noted as arrowheads). At 48 hpf, the otoliths of *nfe2* morphants were normal (Figure 6, panel E), but there was a lack of projections of epithelium forming the semicircular canal system (noted as an arrow in panel B). At 72 hpf, the distance between otoliths had closed in all *nfe2* morphants (Figure 6, panel F) and there was a lack of a semicircular canal system (noted as an arrow in panel C) in most of the morphants (Table 2).

In light of the role of nfe2 in hematopoiesis, an additional set of experiments was performed to examine blood flow in *nfe2* morphants at 48-hpf. This was done using *gata1* ^dsRED^ embryos, which express dsRED in mature erythrocytes [54]. No change in blood flow through the MsV was observed (Table 2).

### Response to Chemical Exposure and Regulation of *nrfs* by Nrf2a and Nrf2b

**Figure 7:**
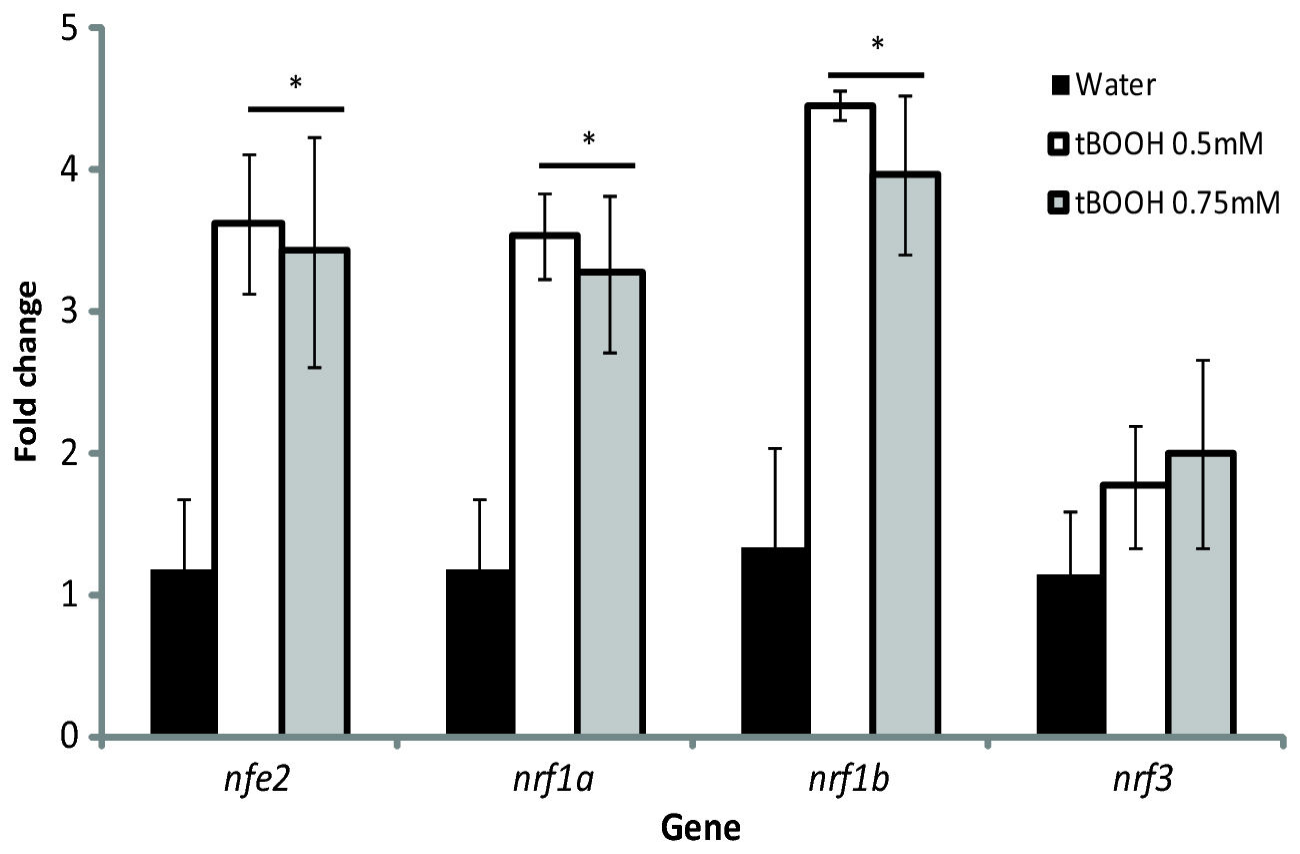
Expression of *nrf* genes following tBOOH exposure at 96 hpf. Larvae were exposed to either water (black bars), 0.5mM tBOOH (white bars), or 0.75mM tBOOH (grey bars) for 6 hours and then sampled. Values were normalized to the water control within each gene and β-actin 1 was used as the housekeeping gene. Fold change data are presented as the mean ± S.E.M. (error bars), and *N* = 3 pools of 25 embryos. Data were analyzed using ANOVA and Fisher's PLSD (*, *p* ≤ 0.05).

To assess the relative responsiveness of nrf genes to oxidative stress, zebrafish embryos were exposed acutely to tBOOH, a pro-oxidant chemical. At 96 hpf, a sublethal exposure to tBOOH (0.5 or 0.75 mM for 6 hr) induced the expression of *nfe2*, *nrf1a*, and *nrf1b* but had no effect on the expression of *nrf3* (Figure 7).

**Figure 8:**
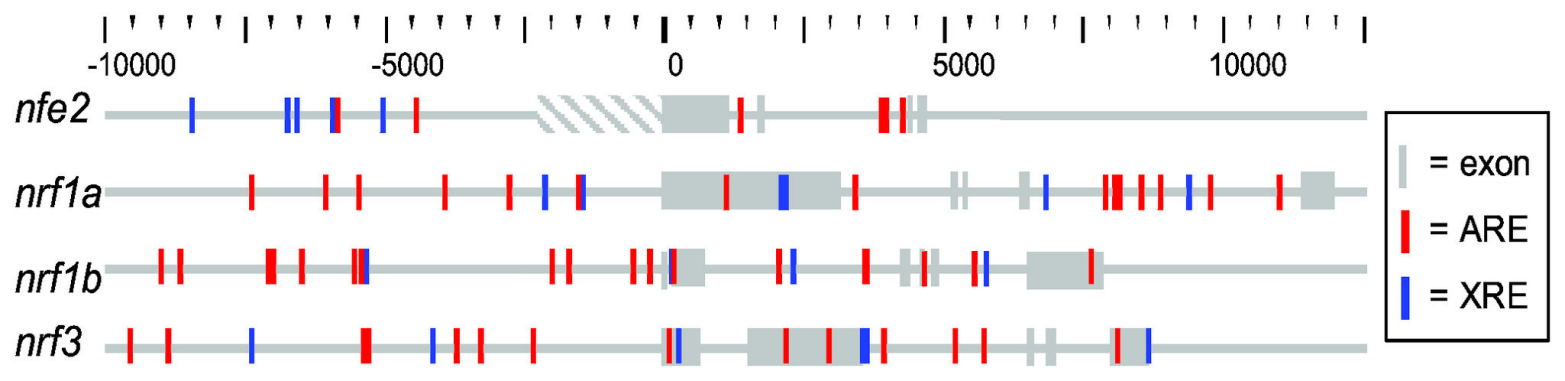
Putative antioxidant response elements (ARE) and xenobiotic response elements (XRE) in the proximal regulatory region of zebrafish *nfe2* and *nfe2*-related (nrf) genes. ARE and XRE positions were mapped using a fuzzy search for the core response elements determined in zebrafish [57,58]. Exon positions were exported from the Ensembl database (Zv9).

**Figure 9:**
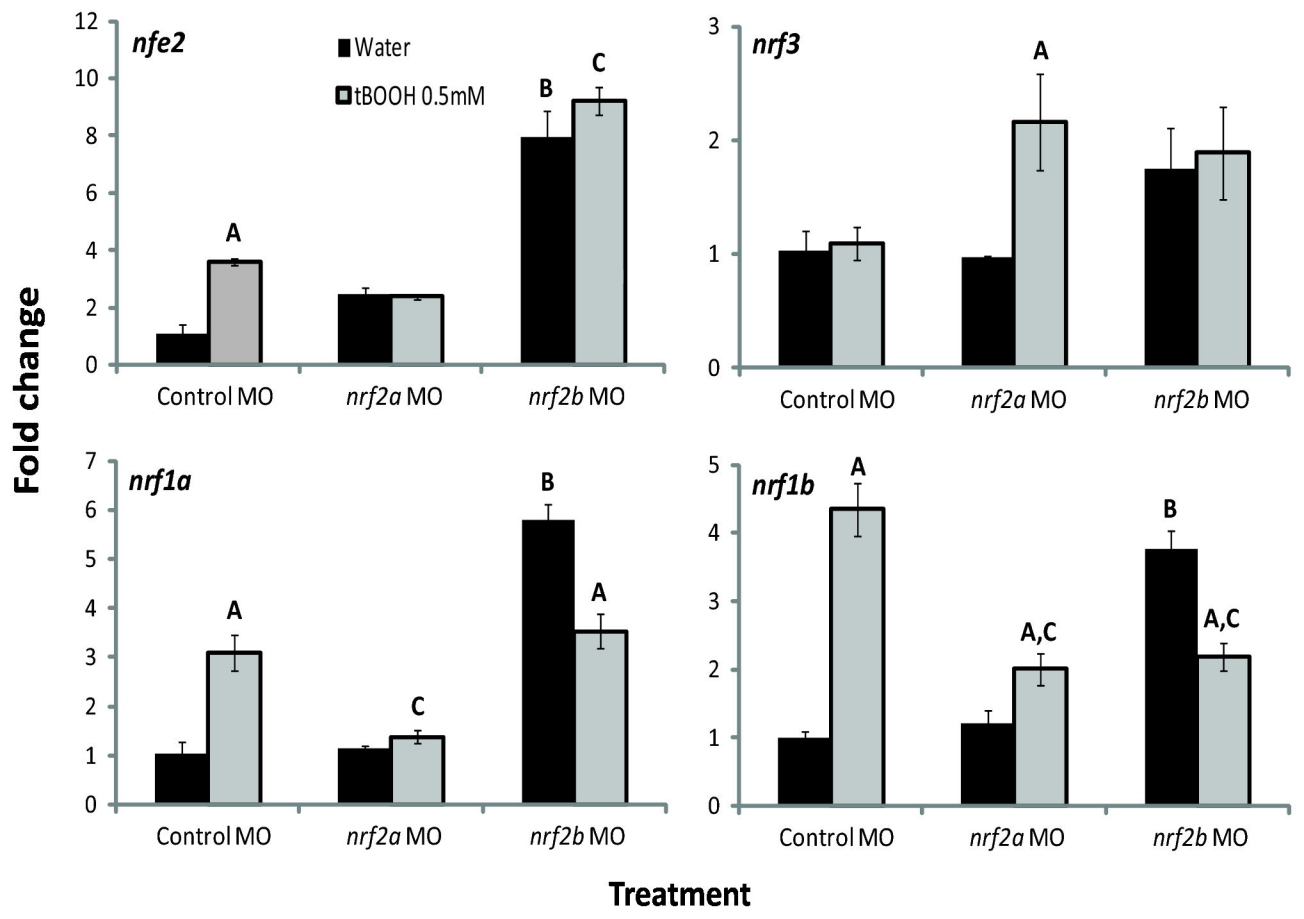
Effects of tBOOH and Nrf2a and Nrf2b morpholino treatment on *nrf* gene expression. Embryos were injected at the one- to four-cell stage with control-MO, *nrf2a*-MO, or *nrf2b*-MO and then treated with either water or 0.5 mM tBOOH for 6 hours starting at 96 hpf. Values were normalized to the control (embryos injected with control-MO and treated with water) within each gene; *β-actin 1* (actb1) was used as the housekeeping gene. Fold change data are presented as the mean ± S.E.M. (error bars), and *N* = 3 pools of 25 embryos. Data were analyzed using ANOVA and Fisher's PLSD (A, difference between water and tBOOH within a MO treatment; B, difference between control MO with water treatment and *nrf2b* MO with water treatment; C, difference control MO with tBOOH treatment and either *nrf2a* or *nrf2b* MO with tBOOH treatment; *p* ≤ 0.05).

Potential antioxidant response elements (AREs) for *nrf* genes were identified *in silico* using the fuzzy search algorithm, fuzznuc [56], to search the 10,000 base pair upstream promoter region and introns of each of the *nrf* genes (Figure 8). Two putative AREs were located in the promoter of *nfe2*, eight in the promoter of *nrf1a*, fourteen in the promoter of *nrf1b* and nine in the promoter of *nrf3*. Given that these are potential binding sites for Nrf2a and Nrf2b, the relative role these two proteins in the direct basal and inducible transcriptional regulation of *nfe2*, *nrf1a*, *nrf1b* and *nrf3* was examined through morpholino knockdown (Figure 9). 

Nrf2a was found to have a role in the induction of *nrf* genes by the oxidant tBOOH. Knockdown of Nrf2a prevented the induction of *nfe2 and nrf1a* observed following exposure to tBOOH. However, with *nrf1b*, Nrf2a knock down inhibited but did not prevent induction. Nrf2a was found to have a role in the expression of *nrf3*. This gene was not inducible by tBOOH alone, but upon simultaneous knockdown of Nrf2a and exposure to tBOOH, expression was significantly increased as compared to that seen with tBOOH or knockdown of Nrf2a alone. 

Nrf2b also was found to have a role in the both the basal and inducible transcription of *nrf* genes. The expression of *nfe2* was significantly greater in Nrf2b morphants, regardless of treatment. In Nrf2b morphants, expression of *nrf1a* and *nrf1b* was significantly higher than in embryos injected with control morpholino. Expression of *nrf1a* and *nrf1b* was decreased in Nrf2b morphants when treated with tBOOH. There was no effect of Nrf2b knockdown on the expression of *nrf3* in either the control or tBOOH treated embryos. 

## Discussion

This report details the first comprehensive study of the temporal expression, knockdown phenotypes and inducibility by oxidative stress of the *nrf* family of CNC transcription factors throughout zebrafish development (Table 3). Expression of the genes in this family varied widely during development and none was found to be essential for viability within the timeframe examined (0-120 hpf). However, Nfe2 knockdown induced a distinct phenotype of a non-functional swim bladder and disorganized cellular structure and morphology in the pneumatic duct and other structures, with nearly 100% penetrance. All members of the *nrf* family, except *nrf3*, were inducible by oxidant exposure. Lastly, the transcriptional regulation of the gene family may involve its own family members, Nrf2a and Nrf2b. Given their broad expression during development, the genes in the *nrf* family may play various, yet undiscovered, roles in development from zygote to larvae. 

**Table 3:** Summary of results.

**Gene**	**Stage of Maximal Developmental Expression**	**Spatial Expression Pattern**	**Effect of Gene Knock-down**	**Induced by tBOOH**	**Role of Nrf2a in regulating Basal Expression?**	**Role of Nrf2a in regulating Inducible Expression?**	**Role of Nrf2b in regulating Basal Expression?**	**Role or Nrf2b in regulating Inducible Expression?**
*nfe2*	Oocyte and Hatching	Intermediate cell mass and circulating blood^1^	Non-functional swim bladder	Yes	No	No	Yes	Yes
*nrf1a*	Larvae	Undetectable^2^	None	Yes	No	Yes	Yes	No
*nrf1b*	Segmentation	No data	None	Yes	No	Yes	Yes	Yes
*nrf2a*	Larvae^3^	Nose, gill, liver, and intestine^4^	None	Yes	No data	No data	No^3^	Yes^3^
*nrf2b*	Hatching^3^	No data	None	Yes	Yes^3^	Yes^3^	No data	No data
*nrf3*	Oocyte	Head region and pectoral fins	None	No	No	Yes	No	No

^1^ [8]

^2^ [62]

^3^ [7]

^4^ [9]

Maximal developmental expression, spatial expression, effect of gene knockdown, inducibility by tBOOH, and regulation by Nrf2a and Nrf2b for each *nrf* gene is summarized.  Results from this paper unless otherwise indicated.

### Temporal and Spatial Expression of *nrf* genes during development

Levels of transcripts for members of the *nrf* family were wide-ranging during development; peak expression was gene-specific and occurred at distinct stages: oocyte, segmentation, hatching and early larvae. Whether these changes in transcript abundance reflect changes in transcription or mRNA stability remains to be determined. In either case, the expression of mRNA from genes during these critical periods implies differing roles during development. *nfe2* and *nrf3* transcripts were at their highest abundance in the unfertilized egg and in the early zygote (Figures 1 and 2). In zebrafish, 17% of all genes have been shown to have maximal transcript abundance at the maternal stage [63]. Of these 17%, most are deposited and presumably translated to accomplish early embryogenesis, but a small proportion, while maternally deposited, are also transcribed by the embryo through later stages in development [64]. The *nrf* genes that are maternally deposited appear to fall into the second category, as their expression persists during development, albeit at much lower abundances than at the zygote stage. 

The persistent expression of the *nrf* genes suggests they have roles in regulating the transcription of key genes throughout development. A known target of mammalian *NFE2* and *NRF3*, *NQO1* [65,66], is present at high levels at the mid-blastula transition (MBT) [67]. NQO1 combats oxidative stress by multiple mechanisms including the two-electron reduction of quinones, preventing generation of reactive oxygen by redox cycling [68]. Additional targets of mammalian NFE2, such as glycogen synthase kinase 3 beta (*GSK3B*) and monoglyceride lipase (MGL) [66], were also increased post-MBT in zebrafish [67]. Whether these genes are targets of Nfe2 and Nrf3 in zebrafish is yet to be determined. 

*In situ* hybridization analysis also provides clues to the potential role of *nfe2* and *nrf3* during later stages of development. The spatial expression of *nfe2, nrf1a*, and *nrf2a* has been previously explored by *in situ* hybridization in developing zebrafish [8,61,62]. However, the expression of *nrf3* in zebrafish is newly described in this paper. *nrf3* transcripts were observed in the head region and pectoral fin buds during segmentation, with expression becoming more specific to the mid hindbrain junction (also known as the isthmus) during the early larvae period (Figure 3). At any given time, the expression of *nrf3* was similar to that of several other genes; however, the trajectory of spatial expression during development, which changes from widespread to very localized, does not match the pattern of any other gene. At 50 hpf in zebrafish, cytochrome P450 1C1 and 1C2 have been shown to similarly localize to the branchiogenic primordia and pectoral fin buds [55]. This localization, however, is dependent on dioxin-induced transactivation of aryl hydrocarbon receptor 2 (Ahr2). For the *in situ* localization assays performed in this study, embryos were not exposed to chemicals. There may, however, still be a link between Ahr2 and spatial regulation of *nrf3*. Several putative dioxin response elements, potential transcription factor binding sites for Ahr2, were found in the promoter and first intron of *nrf3* (Figure 8). Furthermore, Ahr2 has been shown to regulate the basal expression of *nrf2a* and the TCDD-inducible expression of *nrf2b* [7]. The role of Ahr2 in the basal regulation of *nrf3* has yet to be explored.

 In addition to *nrf3*, other genes expressed in the isthmus at 72 hpf include *wnt1* and *pax2* [69]. The timing of gene expression at the isthmus is critical to the development of vertebrate brain architecture. Without an understanding of the cellular regulators and targets for *nrf3* it is hard to speculate about its function, but its expression suggests a possible role in the development of this region. 

*nrf1b* was at its highest abundance in the embryo during segmentation (Figures 1 and 2). This pattern is in contrast to its paralog *nrf1a*, which peaked later, at the early larval stage. The non-overlapping expression patterns suggest that these genes may have undergone subfunctionalization. Their mammalian ortholog, Nrf1, regulates various genes in the ARE-gene battery [70] including NQO1, GCL, and metallothionein 1 [39]. One or both of the Nrf1 paralogs may regulate the expression of some of these target genes, a hypothesis that remains to be tested. In mammals, there is overlap in genes that are regulated by both Nrf1 and Nrf2 via AREs [40]. If the same pattern holds in zebrafish, data from *nrf2a* and *nrf2b* loss-of-function experiments [7] may provide insight into potential *nrf1a* and *nrf1b* gene targets. Genes such as glutathione S-transferase P (*gstp1*) and microphthalmia-associated transcription factor (*mitfa*) are regulated by *nrf2a* in the developing zebrafish [7] and may also be targets of *nrf1a*, whose expression peaks at a similar time point (Figures 1 and 2). 

### Phenotypes of Nrf morphants

Evidence of a potential difference in the developmental role of *nrf1* genes between mammals and zebrafish was discovered in the morpholino knockdown experiments. Nrf1 has been shown to be critical to mammalian development [20]. Since there are two copies of the *nrf1* gene in zebrafish, we used a loss-of-function approach to explore the roles of each gene during development. To our surprise, knockdown of either protein alone or knockdown of both proteins together did not cause embryonic mortality or any gross abnormalities. However, the morpholinos did not completely block the translation of either *nrf1*. To recapitulate the embryonic lethality associated with the mammalian Nrf1 ortholog, it could be that a complete loss-of-function (genomic knockout) would be required. Alternatively, the functions of the genes may have diverged from the mammalian NRF1, such that *nrf1* genes are not essential in zebrafish. In the conditional knockout mouse, where Nrf1 is ablated in the liver, Nrf1 was shown to be critical for regulating liver lipid homeostasis, as well as in the protection against endogenous oxidative stress [71]. Further, in the triple knockout mouse (Nfe2^-/-^: Nrf2^-/-^: Nrf3^-/-^), many individuals survived to adulthood, indicating an overlapping and compensatory role of *Nrf1* within the gene family [22]. Such possibilities have yet to be explored in zebrafish.

Morpholino knockdown of *nrf3* also did not produce any phenotype change. Nrf3 has been linked to carcinogenesis [72], cellular differentiation [73], and inflammation [22]. Non-challenged *Nrf3* knockout mice survive to adulthood and are healthy [22]. However, exposure to benzo[a]pyrene resulted in increased lymphoma and mortality relative to wild-type [72]. While we did not observe any phenotype in the zebrafish embryos treated with the *nrf3* morpholino, as suggested for *nrf1*, we cannot rule out the possibility that we might only see a phenotype with a genomic knockout. It is also possible that morphological or functional phenotypes may be observed if Nrf3 knockdown zebrafish embryos were challenged with toxicants. 

The only knockdown that elicited an obvious phenotype was that of Nfe2. *nfe2* is an important regulator of erythropoiesis in mammals [19,74,75] and reportedly also in zebrafish [8]. Some mice lacking Nfe2 are able to survive to adulthood, but suffer from red blood cell abnormalities (reticulocytosis, hypochromia, target cell formation, dysmorphic cell forms, reduced hemoglobin). Many succumb to perinatal death due to thrombocytopenia. Zebrafish in which Nfe2 was knocked down did not show any obvious hematological phenotypes, but we did observe that at 96 hpf those larvae lacked proper cellular organization in the pneumatic duct, which connects the esophagus to the swim bladder (Figure 5). While the swim bladder was able to be inflated, larvae seemed to lack control of the inflation and deflation mechanism, potentially due to poor functioning as suggested by disorganized epithelial cells, poorly developed mucus cells at the ductal origin, and lack of developed periductal muscles. Grossly, the larvae looked like they lacked an inflated swim bladder (Figure 5B). This swim bladder effect was seen with two independent MOs, suggesting a specific effect. Upon histological examination, air lumens were identified in swim bladders of both *nfe2*-morphant larvae and controls (5C-E), but the ability to expand or retain air in the bladder appeared to differ. Histological examination at additional time points may help to reveal earlier changes and the mechanisms leading to the swim bladder phenotype. 

Poor functioning of the swim bladder and disorganized epithelium in the pneumatic duct have been previously described in cloche (clo) mutants [76]. *Cloche* mutants lack most endothelial cells [77], key components of the circulatory system. Additionally, *clo* mutants also lack *nfe2* expression in the blood [8]. Given these previous results and our findings, it is possible that Nfe2 is a key transcription factor in an endothelial cellular signaling pathway downstream of *cloche* and that the swim bladder defects seen previously in *cloche* mutants [76] may be explained by the loss of *nfe2* expression [8]. Beyond the swim bladder and related organs, cellular disorganization was also observed in other cells in the larvae, namely the gills, cartilage and the mucous cells lining the esophagus. This targeted cellular disruption of particular cell types points to a novel role of Nfe2 in determining cell morphology and organization. Future studies to determine the localization of *nfe2* expression between 48 and 96 hpf may help to better define that role.

There may also be a link between the development and function of the swim bladder and the Ahr pathway. In zebrafish treated with TCDD, a known agonist of Ahr2 and Ahr1b, a dose-dependent incidence of swim bladder inflation failure has been observed [78]. Fish treated with PCB126 also experience an inhibition of swim bladder inflation in an Ahr2-dependent manner [53]. While there were minor phenotypic differences (cell necrosis in the swim bladder was seen in 1/11 larvae treated with PCB126, while we saw no evidence of necrosis), those studies and ours converge on a non-functional swim bladder phenotype. Although the swim bladder phenotype observed in our experiments was caused by the loss of Nfe2 rather than by chemical treatment, Nfe2 could be involved also in the effects of TCDD and PCB-126 mediated through Ahr2. Several putative DREs were found in the promoter and introns of *nfe2* (Figure 8), suggesting possible basal and inducible regulation of this gene by the Ahr family. 

Hedgehog signaling has been implicated in the proper development of otic vesicles, esophagus, and swim bladder [79-82]. While the molecular mechanisms are still poorly understood, zebrafish embryos treated with cyclopamine, a pharmaceutical agent that inhibits hedgehog signaling, leads to improper development of the posterior macula in the otic vesicle [82]. Mutants lacking *Indian hedgehog a* (*ihha* ^*-/-*^) suffer from dysmorphic esophageal and swimbladder epithelium [81], as well as a lack of swimbladder inflation [80]. Differentiation of the esophageal epithelium [79] and swimbladder inflation [80] are also perturbed in another mutant known as *syu*, which is partially deficient in hedgehog signaling due to mutation in the gene *sonic hedgehog a* (*shha*). Comparing images of the defects from these four studies, the otic vesicle and swim bladder phenotypes appear similar to that observed in our study. A preliminary analysis of 10 kb of promoter sequence for mammalian NFE2 binding sites (similar to AREs) was performed to determine if Nfe2 might regulate these hedgehog genes. Putative Nfe2 binding sites were found in *dhh*, *shha, shhb*, *ihha*, *ihhb*, *hhat*, *hhatla*, and *hhatlb* (Table S1). Taken together, these results suggest a possible role of the Nfe2 in regulating morphogenesis of the esophagus and the swim bladder during development, potentially through the hedgehog pathway. It will be of interest to investigate whether there are endogenous activators of nfe2 or nrf2, such as oxidized phospholipids [83,84], that may act as physiological regulators of swim bladder or otic vesicle development.

### *nrf* gene inducibility with oxidative stress and crosstalk with Nrf2a and Nrf2b

Oxidative stress is marked by the disruption of redox signaling and control [85]. Nrf2 activates the transcription of many genes encoding proteins involved in quenching and detoxifying of ROS in the cell [21], and Nrf1 is involved in the regulation of genes in glutathione synthesis and other enzymes related to the oxidative stress response [39,40]. In the zebrafish, the role of *nrf2a* and *nrf2b* in the oxidative stress response has been characterized [7,9,86]. *nrf2a* is essential in regulating the expression of several oxidative stress response genes induced by tBHQ. On the other hand, *nrf2b* may act as a repressor of genes involved in the response. With this in mind, we sought to characterize the expression of the other *nrf* genes in embryos exposed to a pro-oxidant. At 96 hpf, *nfe2*, *nrf1a*, and *nrf1b* were all significantly induced by exposure to tBOOH but tBOOH did not induce *nrf3* gene expression (Figure 7). 

To better understand the regulation of these *nrf* genes, the role of Nrf2a and Nrf2b in that inducible response was explored. Putative AREs were identified in the promoter and first intron of each of the induced genes (Figure 8), possibly acting as transcription factor binding sites for the Nrf2 proteins. Through the use of gene knock down, we found that Nrf2a was essential for the induction of *nrf1a* and *nrf1b* by tBOOH (Figure 9). Conversely, when Nrf2a was knocked down, the expression of *nrf3* was significantly increased upon treatment with tBOOH. A similar pattern was found with *nrf2a* when Nrf2b was knocked down [7]. There was no significant change in the expression of *nfe2* with Nrf2a knockdown, but there was a suggestion of an increase, and tBOOH inducibility was lost. This study provides additional evidence that Nrf2b is a basal transcriptional repressor; the expression of *nfe2*, *nrf1a* and *nrf1b* increased in the control embryos injected with *nrf2b* morpholino. These results demonstrate that there is significant crosstalk within the *nrf* gene family that likely is important to the ability of the developing animal to respond to oxidative stress. 

In conclusion, this paper sheds light on the developmental expression of the entire *nrf* gene family in zebrafish. We show that each of the genes is expressed throughout development, but peak expression is gene-specific and occurs at distinct developmental periods. Spatially, *nrf3* was shown to be expressed in the head region early in development and then became more distinct to the mid hindbrain junction as development progressed. Loss-of-function experiments showed that while none of the *nrf* genes appeared to be essential, *nfe2* was shown to be an important regulator of swim bladder function and cellular morphology in the pneumatic duct and other structures. While much of the attention on the oxidative stress response has focused solely on the Nrf2 proteins, we showed that oxidative stress induces the expression of multiple members the *nrf* gene family. Lastly, we established that crosstalk between Nrf2a and Nrf2b occurs with the remainder of the family, both basally and upon treatment with a pro-oxidant. While much is still to be learned about these genes and their protein products, this study presents novel data that provide a foundation for future research to establish their importance in vertebrate development and the response of embryos to oxidative stress. 

## Supporting Information

Table S1 **Putative NFE2 sites found in hedgehog pathway genes (see Methods).** NFE2 motif taken from Wang *et al*. [59].
(PDF)
