# Supplementary material for: Developmental Expression of the Nfe2-Related Factor (Nrf) Transcription Factor Family in the Zebrafish, Danio rerio
Source: PLoS One. 2013 Oct 24;8(10):e79574. doi: 10.1371/journal.pone.0079574 (PMC3840143; doi:10.1371/journal.pone.0079574)
Supplement: Table S1 — Putative NFE2 sites found in hedgehog pathway genes (see Methods). NFE2 motif taken from Wang et al. [59]. (PDF) [file pone.0079574.s001.pdf]

Table S1. Putative NFE2 sites found in hedgehog pathway genes (see Methods). NFE2 motif taken from Wang *et al.* (2012) Genome Res 22: 1798-1812.

ENSEMBL Gene Model	Gene Name	Relative to Gene Start		score	p-value	q-value	matched sequence
**ENSDARG00000037062**	dhh	-8187	-8169	10.6232	2.58E-05	0.646	ATATATGCATAGTCATAAT
**ENSDARG00000037062**	dhh	-6352	-6370	12.4535	1.17E-05	0.646	TTAGATGCTGCGTCATTCG
**ENSDARG00000074428**	hhat	-3318	-3336	7.52569	8.43E-05	0.823	ATTAGTGATGAGTAATCAT
**ENSDARG00000039051**	hhatla	-3068	-3050	7.52569	8.43E-05	0.823	ATACTTGACAAGTCACGCC
**ENSDARG00000039051**	hhatla	-1935	-1917	9.63761	3.84E-05	0.679	AATATTGCAGTTTCATTGC
**ENSDARG00000039051**	hhatla	-372	-354	7.66649	8.02E-05	0.823	GAACATGCAAACTCACACA
**ENSDARG00000005139**	hhatlb	-9434	-9452	7.2441	9.31E-05	0.823	TACTCAGCTTTGTCATCAC
**ENSDARG00000005139**	hhatlb	-6267	-6285	10.2008	3.07E-05	0.646	GATCCTGATGAATCATCTA
**ENSDARG00000058733**	ihha	-6658	-6640	8.37046	6.21E-05	0.76	AAGATTGCTGAATAACGCA
**ENSDARG00000058815**	ihhb	-2476	-2494	11.3271	1.92E-05	0.646	CAATTTGCTAAATCACACT
**ENSDARG00000058815**	ihhb	-1123	-1141	10.06	3.25E-05	0.646	AGTCTTGCAGAGTCAGACT
**ENSDARG00000068567**	shha	-7090	-7072	10.4824	2.74E-05	0.646	GAAAATGATGTCTCATGAG
**ENSDARG00000068567**	shha	-6721	-6703	7.3849	8.86E-05	0.823	AGAACTGCTAACTTATCCA
**ENSDARG00000068567**	shha	-5067	-5085	8.51126	5.90E-05	0.76	TAGTATGCCAAGTCATTTG
**ENSDARG00000068567**	shha	-3942	-3960	8.93364	5.03E-05	0.76	CGCTGCGATGAGTCACACA
**ENSDARG00000038867**	shhb	-7247	-7229	8.37046	6.21E-05	0.76	TAACATGCTATGTCATAAA
**ENSDARG00000038867**	shhb	-5512	-5494	11.3271	1.92E-05	0.646	ATCTGTGCTGATTCACCAA
**ENSDARG00000038867**	shhb	-1819	-1801	10.6232	2.58E-05	0.646	GTTTTTGCTCACTCATATG
